# Frequency of sexual dysfunction and its causative factors among diabetic women in Turkey

**DOI:** 10.12669/pjms.303.4638

**Published:** 2014

**Authors:** Nuriye Buyukkayaci Duman

**Affiliations:** 1 Nuriye Buyukkayaci Duman,Assistant Professor,Hitit University School of Health,Corum, Turkey.

**Keywords:** Diabetes, Depression, Sexual Dysfunction, Causative Factors

## Abstract

*** Objective: ***To determine the frequency of sexual dysfunction and its causative factors among the diabetic women.

***Methods:*** The sample of the research was made up of 200 diabetic women selected using simple-random sampling who went to endocrinology poly clinics for counseling and treatment. The data were gathered with Data Collection Form for Women’s Descriptive Characteristics designed by the researchers using the information in literature, Female Sexual Function Index and Beck Depression Inventory. The data were assessed with percentages, arithmetic means, standard deviation and ANOVA test in computer environment.

***Results:***Out of two hundred diabetic patients nearly halfof the participant women (48.0%) underwent depression and nearly one in four women experienced sexual dysfunction (26.2%). In the analysis, there was statistically significant correlation between women’s sexual dysfunction and diabetes type, diabetes complications, HbA1C value, having a sexual problem and presence of depression.

***Conclusion: ***This study showed that one in two diabetic women suffered from depression while nearly one in four diabetic women expereienced sexual dysfunction.

## INTRODUCTION

Diabetes Mellitus (DM) is a chronic systemic disease in which carbohydrate, protein and fat metabolisms are deteriorated due to a complete or partial insulin hormone deficiency.^1^ DM may lead to organ dysfunctions or organ losses because of micro / macro vascular complications. One of the major problems caused by DM is sexual dysfunction (SD).^2^ DM is one of the etiological factors leading to sexual dysfunctions; patho physiologically related to neurogenic, psychogenic and vascular problems.^2^^,^^3^

The studies on this issue indicate that people with DM experience more SD than those without DM.^2^^-^^4^ When the DM is examined in terms of its effect upon sexual dysfunction, it is seen that studies mainly focused on male SD while there are limited number of studies on diabetic women’s sexual problems.^5^^-^^11^ However, the studies point out that DM affects female sexuality negatively, too.^5^^-^^11^ SD prevalence among diabetic women ranges between 15% and 80%. The commonly suffered sexual problems among women with DM are poor sexual arousal or sexual arousal with slow lubrication and lack of sexual desire.^5^^-^^11^There are limited number of studies on the effect of diabetes upon female sexual function, therefore this study was study conducted in order to determine the frequency of sexual dysfunction among diabetic women and to discover the causative factors contributing to sexual dysfunction among women.

## METHODS

The population of the research was consisted of women who came to Hitit University Education and Research Policlinics for endocrinology treatment between March 2012 and August 2012. The sample of the research was made up of 200 eligible women selected using simple-random sampling between these periods.

The data were gathered with Data Collection Form for Descriptive Characteristics of Diabetic Women, Female Sexual Function Index and Beck Depression Inventory by the researchers using the information in literature.

***Female Sexual Function Index (FSFI):***Female Sexual Function Index was developed by Rosen et al. (2000) in order to measure sexual function of the women who are clinically diagnosed with sexual arousal dysfunction.^12^ Cronbach Alpha coefficients were separately analyzed for six domains and the results ranged from 0.89 and 0.97. Female Sexual Function Index assesses sexual function problems or sexual problems that occur during the last four weeks. There are six domains: desire, arousal,lubrication, orgasm, satisfaction and pain. The highest raw score that can be obtained from the scale is 95 whereas the lowest raw score is 4. The highest score of the scale is 36 whereas the lowest score is 2; which are obtained by multiplying mean domain scores by factor loads. Cut-off point is recommended as 26 and those who have scores ≤ 26 are regarded as having sexual dysfunction. In our research, too, cut-off point was 26.

***Beck Depression Inventory (BDI): ***BDI, developed by Beck (1960), is one of the most used inventories in clinics and researches and can be administered for individuals aged between 13 and 80.^13^ The scale consist of 21 statements and is used to objectively measure the degree of depression and physical, emotional, mental and motivational symptoms seen during depression. Scores for the statements of the inventory range from 0 to 3. All the scores are added and depression score is obtained. The highest score of the inventory is 63 (21 x 3). A higher total score means a higher level or severity of depression. Scores obtained from the inventory can be evaluated as follows:

***Score Evaluation:***

0-9 Normal

10-15 Slight depression

16-23 Moderate depression

24-63 Severe depression

**Figure 1:**
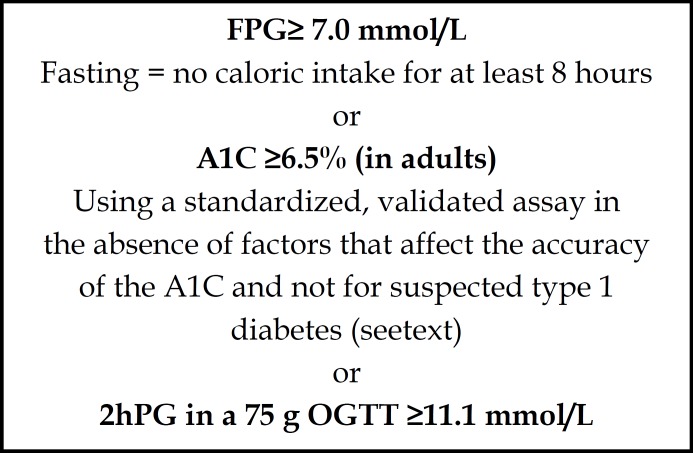
Diagnosis of diabetes

***Evaluation of the Data: ***In the study; for the women, first fasting plasmaglucose (FPG), HbA1c was administered and then 75 gr Oral Glucose Tolerance Test (OGTT) was administered by using the diagnosis criteria recommended by the American Congress of Obstetrıcıans and Gynecologısts (ACOG, 2001). ^20^According to the test results; women whose one or more test results were higher were accepted as GDM (Figure 1.). 

The data obtained were analyzed with SPSS 17.0 statistical package program. The data were assessed with percentages, arithmetic means and standard deviation and chi-square test in computer environment.

***Ethical Considerations of the Research:***The ethical suitability of the research was approved by ethical council (23/24). All patients were informed of the purpose the study with written documents and were told that the information would not be disclosed and their oral consents were obtained. Thus, those volunteer for the research were included in the study.

## RESULTS

A total of two hundred women participated in the study, 60% were aged between 29 and 34 , had high-school degree (67.5%) and worked (59.5%). Nearly one of the two women had a total monthly income between 1001 and1501 $. All the women were married and nearly three of the five women had marriage duration between 5 and 10 years. Also, nearly all the women had children (Table-I).

According to the findings relating to health history of the women, most of the women suffered from Type 2 diabetes and used medicines which included oral antidiabetics (60.0%) and insulin (40.0%). Four of the five women had diagnosis duration fewer than five years and HbA1C was between 6 and 7 (80.0%). In the light of these findings, most of the women did not develop any diabetic related complication (92.5%). Women with diabetic complication had vaginal moniliasis (80.0%), frequent urinary tract infections (75.6%) and one women developed retinopathy (Table-II).

When the findings relating to the women’s depression history were examined, it was found that according to the BDI scores nearly one of the two women had depression symptoms (48.5%). Nearly all of the women with depression had slight depression (95.8%) while only two women had moderate depression (Table-II).

According to the mean FSFI scores; the most affected sexual domains were generally arousal (20.5%), orgasm (19.5%), lubrication (18.8%) and satisfaction (17.5%). According to FSFI cut-off point 26.2% of the women had SD (sexual dysfunction). The most affected domains were arousal (23.4%), desire (22.3%) and orgasm (20.2%) among the women with SD (Table-III).

It was explored that there was a positive correlation between type of diabetes, diagnosis duration, HbA1C level, presence of diabetic complication and use of medicine, and SD. According to the findings, it was understood that SD was seen more among the women who had Type 1 diabetes, had diabetes for more than 10 years, developed diabetic complication and did not use medicine (Table-IV). Besides, it was noted that women with depression suffered from SD more than other women. According to the analysis; the correlation between type of diabetes, diagnosis duration, HbA1C level, presence of diabetic complication and use of medicine, and SD was statistically significant (p< 0.05) while the correlation between age, educational status, total monthly income, employment status, marriage duration and having achild and SD was statistically insignificant (p>0.05) (Table-IV).

**Table-I:** Distributions about Some of Women’s Demographic Characteristics

*Characteristics*	*N*	*%*
*Age ( Years)*		
29-34 35-39	80 120	40.0 60.0
*Educational Status*		
Secondary School High School and above	135 65	67.5 32.5
*Professional Status*		
Yes No	119 81	59.5 40.5
*Total Monthly Income* *(Dollars)*		
1001-1501 1501- 2001 2002 veüzeri	110 50 40	55.0 25.0 20.0
*Marital Status*		
Yes No	200 0	100.0 0.0
*Marriage Duration ( Years)*		
5- 10 11-16	130 70	65.0 35.0
*Having a Birth*		
Yes No	180 20	90.0 10.0
Total	200.0	100.0

**Table-II:** Characteristics Related to Diabetic Women’s Health Histories

*Characteristics*	*N*	*%*
*Diabetes Type*	*N=200*	
Type1 Type 2	20 180	10.0 90.0
*The Time of Diagnosis* *( Years)*	*N=200*	
<5 5-10 10>	160 20 20	80.0 10.0 10.0
*Experiencing diabetes complications*	*N=200*	
Experiencing Not Experiencing	15 185	7.5 92.5
*HbA1C Value (%)*	*N=200*	
6-7 7,1-8 8,1-9	160 20 20	80.0 10.0 10.0
*Status of drug use*		
Using Not Using	190 10	95.0 5.0
*Experiencing Depression *	*N=200*	
Experiencing Not Experiencing	96 94	48.0 52.0
*Level of Depression*	n= 96*	
Mild Depression Moderate Depression Severe Depression	94 2 0	95.8 2.1 0.0

* Only those women who had depression.

**Table-III:** Sexual Function Characteristics of Diabetic Women

*FSFI*	*Mean ± SD*	*Min-Max*	*%*
	*N=200*		
Desire Arousal Lubrication Satisfaction Pain Orgasm	3,7570±,14234 3,8070±,14356 4,2260±,14678 4,4430±,14897 4,4570±,13456 4,0380±,14456	1,20-6,00 ,00-6,00 ,00-6,00 ,00-6,00 ,80-6,00 ,00-6,00	12.3 20.5 18.8 17.5 16.4 19.5
*Total Sexual Dysfunction*	*26,9090±,72340*	*2,00-35,70*	*26.2*
*FSFI of the women with sexual dysfunction*	n=53*		
Desire Arousal Lubrication Satisfaction Pain Orgasm	2,3456±,16713 2,3460±,17069 3,2891±,20103 3,5478±,19678 3,6350±,18201 3,4567±,18788	1,20-5,40 ,00-5,70 ,00-5,70 ,00-5,60 ,80-6,00 ,00-6,00	22.3 23.4 15.0 20.2 16.7 4.4
*Total Sexual Dysfunction*	*19,7567±,92456*	*2,00-26,00*	*100.0*

**Table-IV:** Women’s Sexual Dysfunction (SD)according to some characteristics

*Characteristics*	*Experiencing* *SD(%)*		*(%)*	*Chi Square*	*P*
	*Experiencing*	*Not Experiencing*			
*Age ( Years)*					
29-34 34-39	52.0 54.1	48.0 45.9	100.0 100.0	0.897	0.778
*Educational Status*					
Secondary School High School and above	45.0 47.3	55.0 42.3	100.0 100.0	0.756	0.639
*Professional Status*					
Yes No	33.5 38.9	66.5 61.1	100.0 100.0	0.889	0.979
*Total Monthly Income* *( Dollars)*					
1001-1501 1501- 2001 2002 and above	42.0 43.2 40.0	58.0 56.8 60.0	100.0 100.0 100.0	1.003	0.688
*Marriage Duration ( Years)*					
5- 10 11-16	56.2 57.0	43.8 43.0	100.0 100.0	0.858	0.654
*Having a Child*					
Having Not Having	40.3 38.9	59.7 61.1	100.0 100.0	0.889	0.780
*DiabetesType*					
Type1 Type 2	55.0 35.0	45.0 65.0	100.0 100.0	10.285	0.269
*The Time of Diagnosis* *( Years)*					
<5 5-10 10>	32.0 48.0 55.5	68.0 52.0 44.5	100.0 100.0 100.0	8.321	0.465
*Experiencing diabetes complications*					
Experiencing Not Experiencing	32.7 45.4	67.3 54.6	100.0 100.0	9.654	0.389
*HbA1C Value (%)*					
6-7 7,1-8 8,1-9	32.3 45.6 56.4	67.7 54.4 53.6	100.0 100.0 100.0	10.766	0.287
*Status of drug use*					
Using Not Using	35.3 48.2	64.7 51.8	100.0 100.0	5.765	0.453
*Experiencing Depression *					
Experiencing Not Experiencing	56.0 34.2	44.0 65.8	100.0 100.0	8.435	0.342

## DISCUSSION

DM leads to sexual dysfunction among women and men affecting genital organs and many other systems with which these organs function in a coordinated way.^1^^-^^3^ The studies on this issue indicate that diabetic women are at risk as much as men in terms of sexual dysfunction.^5^^-^^11^ When the literature is reviewed, it is seen that there are different findings in relation with SD prevalence among diabetic women. In the study of Enzlin et al. (2002), SD prevalence among Type I diabetic women was by 27% while it was 15% among the women without Type I diabetes.^7^In the study of Yildiz (2008), it was noted that 54.4% of the women with diabetes had SD.^11^ But in our study; according to the mean FSFI scores obtained by the diabetic women 26.2% of them had SD; which was in agreement with the findings of the study of Enzlin et al. (2002).^7^

DM causes ischemia and vaginal vascular insufficiency syndrome due to arteriosclerosis of iliohypogastric/pudendal artery bed. This syndrome increases the risk of vaginal dryness and dyspareunia among the diabetic women.^14^ Besides; diabetic women experience lack of sexual desire and orgasm problems more as compared with the population.^7^^,^^10^^,^^11^ In the study of Enzlin et al. (2002); the most commonly suffered sexual problems experienced by Type I diabetic women were insufficient and/or slow vaginal lubrication and decrease in sexual arousal.^7^ In the study of Yildiz (2008), the most affected areas of the diabetic women were sexual desire, sexual arousal, dyspareunia and sexual satisfaction; respectively.^11^ In our study; according to the mean FSFI scores the most affected areas of the diabetic women were sexual arousal (20.5%); orgasm(19.5%), lubrication (18.8%)and sexual satisfaction (17.5%). The most affected areas of the women with SD were sexual arousal (23.4%), sexual desire (22.3%) and orgasm (20.2%). These findings are similar to those reported in the literature.

DM causes female sexual dysfunction due to vascular, neurologic, endocrinal, psychogenic factors or their combinations.^2^ Particularly old age, long diabetes duration, high HbA1C, poorly controlled diabetes, chronic complications and psychological problems such as anxiety and depression contribute to the development of SD among the diabetic women.^5^^-^^11^

In literature; it is argued that SD risk increases with age in addition to the decreased functioning capacity of genitourinary organs and systems and hormonal changes. The relevant studies emphasize that age is a significant risk factor for female SD. In these studies, it has been shown that SD prevalence is higher among the women in post- menopausal period and old age than those women who belong to young age groups.^4^^,^^15^

In our study; it was noted that there was a positive correlation between body mass index and female SD (p<0.05). The study of Enzlin et al. (2003) reported no correlation between BMI and SD whereas the studies of Dilek (2007) and Yildiz (2008) demonstrated a positive correlation between BMI and female SD.^3^^,^^10^^,^^11^ These findings were in line with our findings.

In literature, it is stated that type of diabetes is the most important factor that affects female SD.^6^^,^^10^^,^^11^ In our study; SD prevalence in Type I diabetic women (55.0%)was higher than Type II diabetic women (35.0%)..^6^^,^^10^Unlike our study; in the study of Yildiz it was seen that nearly 8 of the 10 women with SD had Type II diabetes.^11^ It was understood that the relevant studies pointed out different results; which may have resulted from the fact that the studies were conducted with different groups in terms of type of diabetes, sample size and diagnosis duration.

Prolonged hyperglycemia increases the risk for peripheral neuropathy. Therefore, if metabolic control of blood glucose level is not ensured as the diabetes duration prolongs; female SD risk increases.^5^^,^^6^^,^^9^^,^^14^In our study; it was discovered that a positive correlation existed between diabetes duration and SD (p<0.05). As the diabetes duration prolonged, so did SD prevalence. Our findings were similar to those reported in literature.

We observed that there was a positive correlation between HbA1c level, development of diabetic complications and drug use, and SD (p< 0.05). To put it differently, SD was seen more among the women with diabetic complication (45.4%) than those without diabetic complication (32.7%) and among the women who did not use medicines (48.2%) than those who used medicines (35.3%). In our study, it was seen that as the HbA1c level went up so did SD prevalence. As far as these findings were concerned; it might be suggested that metabolic control of diabetes had a decreasing effect upon female SD risk. In the studies of Erol et al. Enzlin et al. and Dilek; no correlation was found between HbA1c level and female SD while the study of Yildiz (2008) reported higher level of HbA1c and poorly controlled diabetes was crucial risk factors for female SD; which was similar to our findings.^3^^,^^8^^,^^10^^,^^11^

The most commonly encountered diabetic complications among women in our study were vaginal moniliasis (80.0%) and recurrent urinary system infections (75.6%). According to the literature; vaginal moniliasis and urinary system infections are frequently seen among diabetic women- cause vaginal dryness and dyspareunia and thus increase SD risk.^5^^,^^6^^,^^9^^,^^14^Among the women with recurrent bacterial cystitis; risk for dyspareunia and sexual arousal disorder increases.^16^ In the study of Yildiz ;it was reported that SD developed ten times more among the women who had itching in the genital area.^11^ Our study finding was in line with the literature.

We observed that depression affected female SD development positively. Similar to our study result; the study of Moreira et al. reported that women with depression suffered sexual desire dysfunction more as compared to those women without depression and the study of Speer et al. proved that sexual desire increased as the depression level decreased.^17^^,^^18^However; in the study of Enzlin et al. it was detected that women with SD had higher level of depression than those without SD.^3^ We discovered that such psycho-social factors as educational level, total monthly income, having a job, marriage duration and having child did not affect female SD (p>0.05).

When the relevant studies were investigated, it was seen that there were different study findings. Similar to our study result, the study of Duman & Kocak showed that educational level, total monthly income, having a job, marriage duration did not affect female SD.^19^Likewise; it was reported in the studies of Lewis et al. and Yildiz that educational status and economic status did not affect female SD.^4^^,^^11^Unlike our study; in the studies of Lauman et al. and Dilek ; it was concluded that educational status and having a job affected female SD.^10^ We were of the opinion that the difference among the study findings resulted from the fact that these studies were carried out with women withdifferent cultural features, different health histories and different age groups.

## CONCLUSION

This study showed that one of the four diabetic women suffered from SD. The factors that contributed to SD were type of diabetes, diagnosis duration, use of medicine, HbA1C level, presence of diabetic complication and depression. We recommend that :

1. Counseling departments and family therapy units about sexual issues should be established within endocrine/diabetes clinics.
2. Future studies should be made with randomized controlled groups and with a larger sample size.
